# A novel approach for the co-delivery of 5-fluorouracil and everolimus for breast cancer combination therapy: stimuli-responsive chitosan hydrogel embedded with mesoporous silica nanoparticles

**DOI:** 10.1186/s12967-025-06396-4

**Published:** 2025-03-31

**Authors:** Pooria Mohammadi Arvejeh, Fatemeh Amini Chermahini, Francesco Marincola, Fatemeh Taheri, Seyed Abbas Mirzaei, Akram Alizadeh, Fatemeh Deris, Raziyeh Jafari, Niloufar Amiri, Amin Soltani, Elham Bijad, Ebrahim Soleiman Dehkordi, Pegah Khosravian

**Affiliations:** 1 Cellular and Molecular Research Center, Basic Health Sciences Institute, Shahrekord University of Medical Sciences, Shahrekord, Iran; 2 Student Research Committee, Shahrekord University of Medical Sciences, Shahrekord, Iran; 3 Translational and Advanced Medicine (TAM) Biosciences, Nashville, TN USA; 4 Department of Pathology, Hematology & Anatomical Sciences, School of Medicine, Shahrekord University of Medical Sciences, Shahrekord, Iran; 5 Department of Medical Biotechnology, School of Advanced Technologies, Shahrekord University of Medical Sciences, Shahrekord, Iran; 6 Department of Tissue Engineering and Applied Cell Sciences, Faculty of Medicine, Semnan University of Medical Sciences, Semnan, Iran; 7 Department of Epidemiology and Biostatistics, Shahrekord University of Medical Sciences, Shahrekord, Iran; 8 Medical Plants Research Center, Basic Health Sciences Institute, Shahrekord University of Medical Sciences, Shahrekord, Iran

**Keywords:** Drug delivery, Nanocomposite, Mesopores, Hydrogels, Synergism, Breast neoplasm

## Abstract

**Background:**

Breast cancer remains one of the leading causes of death among women globally, with traditional therapies often limited by challenges such as drug resistance and significant side effects. Combination therapies, coupled with nanotechnology-based co-delivery systems, offer enhanced efficacy by targeting multiple pathways in cancer progression. In this study, we developed an injectable, stimuli-responsive nanosystem using a chitosan hydrogel embedded with mesoporous silica nanoparticles for the co-administration of 5-fluorouracil and everolimus. This approach aims to optimize controlled drug release, enhance the synergistic anticancer effect, and overcome challenges associated with co-loading different therapeutic agents.

**Methods:**

Various techniques were employed to characterize the nanoparticles and the hydrogel. Cell uptake, apoptosis, and proliferation of 4T1 breast cancer cells were evaluated by flow cytometry and Resazurin assay, respectively. The Balb/C mice model of breast cancer, which received the therapeutical nanoplatforms subcutaneously near the tumoral region was used to examine tumor size and lung metastases.

**Results:**

The results revealed that the nanoparticles had a suitable loading capacity and high cellular uptake. The drug release was pH-sensitive and synergistic. By incorporating nanoparticles into the hydrogel, the cell death rate and apoptosis of 4T1 breast cancer cells increased significantly, due to the synergistic effects of co-delivered drugs. Additionally, the combination treatment groups showed a significant reduction in tumor size and lung metastasis compared to the monotherapy and control groups.

**Conclusions:**

These findings underscore the potential of the nanocomposite used to develop a novel co-delivery system to enhance therapeutic outcomes, reduce side effects, and provide a promising new strategy for future cancer treatments.

**Supplementary Information:**

The online version contains supplementary material available at 10.1186/s12967-025-06396-4.

## Introduction

Breast cancer remains a leading cause of mortality among women worldwide [1]. The disease arises from genetic mutations that promote uncontrolled cell proliferation [2–4]. Despite advancements in chemotherapy and radiation therapy, drug resistance and toxicity continue to hinder treatment efficacy, necessitating novel therapeutic approaches.

Nanotechnology has revolutionized cancer therapy, particularly in combination treatments, where co-delivery of multiple drugs enhances therapeutic efficacy while reducing side effects [5]. Nanocarriers improve drug solubility [6, 7] and address physicochemical and pharmacodynamic challenges associated with conventional therapies [5, 8]. Among the most promising strategies is the co-encapsulation of multiple drugs within a single nanoparticle, optimizing synergistic effects [9, 10]. However, challenges such as inconsistent drug loading, stability issues, and misaligned release kinetics can compromise treatment efficacy [11, 12]. Additionally, smaller nanoparticles exhibit better tumor penetration but lower drug-loading capacities, creating a size-efficiency dilemma [11]. Ensuring stability in biological environments and overcoming translational barriers from preclinical to clinical applications remain critical challenges [11, 12].

As researchers continue to refine nanotechnology for cancer therapy, nanocomposites—nanoparticles embedded within a matrix material—have gained attention for their role in combinatorial drug delivery [13–17]. These systems offer several advantages over conventional drug delivery mechanisms, including the ability to simultaneously deliver multiple drugs with complementary mechanisms of action. Additionally, nanocomposites can reduce drug exposure to healthy tissues, overcome drug resistance, and facilitate stimulus-responsive drug release, ensuring targeted and efficient therapy [7, 18–26]. Hydrogels, particularly chitosan (CS) hydrogels (CSHs), enable sustained drug release, minimizing dosing frequency and enhancing patient compliance [27–30]. Another critical component, mesoporous silica nanoparticles (MSNs), exhibit high surface-area-to-volume ratios [31], making them ideal for high drug loading [32, 33], enhanced stability [34, 35], and controlled release [34, 36]. Moreover, MSNs can be easily functionalized to improve drug targeting and therapeutic efficacy [37, 38]. These multifunctional nanoparticles hold great potential for multi-target drug therapies, paving the way for their clinical application in cancer treatment [39].

A well-studied combination in oncology is 5-Fluorouracil (5FU) and everolimus (EVE), both FDA-approved for breast cancer treatment. 5FU inhibits DNA synthesis, while EVE targets the mammalian target of rapamycin (mTOR) pathway, frequently dysregulated in breast cancer [40, 41]. Studies have demonstrated the synergistic potential of this combination in inducing apoptosis and inhibiting tumor angiogenesis [40, 41]. However, despite extensive research on 5FU-based MSN co-delivery systems, no studies have specifically investigated the co-delivery of 5FU and EVE via this nanoplatform.

To address this gap, we developed a controlled-release system utilizing CSHs embedded with MSNs for the co-delivery of 5FU and EVE (MSN/5FU-EVE@CSH). By separately loading each drug into nanoparticles, this approach enhances therapeutic synergy while overcoming the limitations of combination chemotherapy. This novel strategy represents a significant advancement in nanomedicine, providing a sophisticated means to improve cancer treatment outcomes.

## Methods and materials

### Materials

All the chemicals used for the synthesis of MSNs and the assessment of their cellular uptake, namely N-cetyltrimethylammonium bromide (CTAB), NaOH, Tetraethylorthosilicate (TEOS), Mesitylene, 1,1’-dioctadecyl-3,3,3’3’-tetramethylindocarbocyanine perchlorate (DiL) fluorescent dye, 4,6-diamidino-2-phenylindole (DAPI), and paraformaldehyde, were obtained from Sigma-Aldrich (Munich, Germany). Low molecular weight CS, β-glycerol phosphate (β-GP) disodium salt pentahydrate, and hydrochloric acid (HCL), which were used for the preparation of hydrogel, were also sourced from the same company. Additionally, Sigma-Aldrich supplied Trypan blue, resazurin, 5fu powder, dimethyl sulfoxide (DMSO), ketamine, Xylazine, and glutaraldehyde. Roswell Park Memorial Institute medium (RPMI-1640), fetal bovine serum (FBS), and penicillin/streptomycin were purchased from Gibco (Thermo Fisher Scientific, USA), while Annexin V-FITC/PI apoptosis detection kit was acquired from BD Biosciences (San Jose, CA, USA). EVE powder or RAD001 was provided by Novartis (Basel, Switzerland).

### Preparation and characterization of nanocomposite

#### Preparation of MSNs using the sol-gel method

The preparation of mesitylene-MSNs was based on the sol-gel method as explained before [42]. Briefly, a surfactant solution was prepared by dissolving 1 g of CTAB in 480 ml of deionized water and adding 3.5 ml of 2 mol/L (M) NaOH. The solution was stirred at 1000 rpm and heated until it reached 50 °C, when 7 ml of mesitylene was added, and the mixture was agitated for 5 h at this temperature until it reached 80 °C. Then, 5 ml of TEOS was added dropwise and the reaction was continued for 2 h. The resulting nanoparticles were washed several times with deionized water and ethanol and then calcined at 540 °C in a furnace (AFE1800L, Atra, Iran) to remove the CTAB.

#### Preparation of drug-loaded MSNs

To prepare each sample, 50 mg of calcined MSNs were measured and separately mixed with 3 mL of EVE (5 mg/mL concentration) and 5FU (30 mg/mL concentration) stock solutions. The final volume was adjusted to 5 ml with methanol. The mixtures were kept at room temperature and in the dark for overnight stirring. The amount of drugs loaded into the nanoparticles was evaluated by UV–Vis spectrophotometry of the supernatant. The following formulas were used to calculate the loading efficiency (LE) and capacity (LC) of the drugs:

LE% = % (Initial amount of drug– Amount of drug in supernatant) / (Initial amount of drug).

LC% = % (Initial amount of drug– Amount of drug in supernatant) / (Loaded nanoparticles).

#### Hydrogel preparation and encapsulating MSNs

A 2% (w/w) CS solution was prepared by dissolving CS powder in 0.1 M HCL and stirring magnetically overnight at room temperature. Then, a suitable amount of MSNs containing equal concentrations of each drug for the in vitro and in vivo experiments were sonicated and dispersed in the CS solution. The GP solution was obtained by dissolving 1.5 g of β-GP powder in 3 ml of deionized water. After cooling both solutions at 4 °C for 10 min, the GP solution was added dropwise to the CS solution while stirring at 4 °C to produce a clear and homogeneous CS-GP solution.

#### Morphological analysis of MSNs and CSH

The MSNs and CSH were lyophilized after being quickly frozen at -20 °C. The dried sample was coated with gold and its morphological structure was examined by field emission scanning electron microscopy (FE-SEM, Mira, Czech Republic). Additionally, transmission electron microscopy (TEM) images of MSNs were acquired on a 200 kV Schottky field emitter HR-TEM (Hitachi Naka, Japan). The samples for TEM analysis were prepared by adding one drop of the samples on carbon-coated copper grids.

#### MSN and CSH characterization

The hydrodynamic diameter of the particles was measured by dynamic light scattering (DLS; ZEN3600) at 25 °C and 90° angle. The zeta potential of the synthesized MSNs was determined by Zetasizer (Malvern Instruments, Malvern, UK). Nitrogen adsorption-desorption isotherms were obtained by the surface area and porosity analyzer (Quantachrome NOVA Automated Gas Sorption Device, 2000e, USA) at -195.8 °C under continuous adsorption conditions. The surface area, pore size, and pore volume were calculated by Brunauer, Emmett, and Teller (BET) and Barrett, Joyner and Halenda (BJH) analyses. The crystalline structure of the particles was verified by X-Ray Diffraction (XRD) patterns obtained from Nano-Viewer (STOE & Cie GmbH, Germany).

#### Fourier transform infrared spectroscopy (FTIR)

FT-IR spectroscopy was used to investigate the chemical interactions between the functional groups of the hydrogel components and NPs. The spectra of lyophilized free-drug MSNs, drug-loaded MSNs (MSN/drugs), CS powder, β-GP powder, crosslinked CS and finally crosslinked CS encapsulated drug-loaded MSNs (MSN/drugs@CSH) were obtained using a Nicolet Magna IR-550 spectrophotometer (Thermo/Nicolet, Waltham, MA, USA) in the range of 475 to 4000 cm^− 1^.

#### Gelation time

Two different vials were filled with 2 ml of GP-CS and 2 ml of GP-CS embedded with drug-loaded MSNs, respectively. The vials were stored at 4 °C for 12 h to eliminate the bubbles. Then, the vials were put in a 37 °C bath and tilted horizontally every 20 s. The gelation time was the time when the solution solidified completely.

#### Swelling ratio

The hydrogels were sliced into identical shapes and sizes after freeze-drying, and their weight was measured as M_0_. They were placed in centrifuge tubes with 25 ml of PBS shaken at 37 °C and 100 rpm. At the predetermined time, the hydrogels were removed and any surface moisture was absorbed by filter paper and weighted. When they reached the highest weight and stopped swelling, their weight was measured again as M, and the swelling rate was calculated by the following equation.

(M-M_0_) / M_0_ × 100.

#### In vitro release

To evaluate the release of drugs from MSNs and MSN@CSH, two different experiments were conducted. In each experiment, drug-loaded MSNs (MSN/drugs) and MSN/drugs@CSH floated in 2 ml of phosphate buffer at pH 5 and 7.4 and kept in different closed dialysis bags, which were immersed in 50 ml of phosphate buffer with 0.5% tween-80. The experiments were carried out in a shaker incubator at 37 °C and 100 RPM. The dialysis bags had a molecular weight cutoff of 12,000 Daltons. At predetermined intervals, the buffer was sampled and replenished with fresh buffer to maintain sink conditions, which were verified prior to the release studies. The concentrations of 5FU and EVE in the samples were measured based on preprepared standard curves using UV spectrophotometry, with 5FU quantified at a wavelength of 266 nm and EVE at 278 nm.

### Cellular experiments

#### Cell culture

The 4T1 cells were cultured in RPMI-1640, supplemented with 10% FBS and 1% penicillin/streptomycin in the incubator under standard conditions of 37 °C and 5% CO2.

#### Cellular uptake

To evaluate the cellular uptake, DiL fluorescent dye was used to be loaded in the nanosystem. The cellular uptake of free-DiL and MSN/DiL@CSH was assessed and compared statistically in 4T1 cells. The cells were seeded in six-well plates at a density of 2 × 10^5^ cells per well and incubated for 24 h. Then, for 4 h, the cells were treated with 0.1 µg/mL free-DiL and MSN/DiL@CSH with equal DiL concentration. The cells were then detached with trypsin and washed with PBS twice. The fluorescence intensity of the cells was measured by flow cytometry (BD LSRFortessa, BD Biosciences) and analyzed by Flowjo software version 10 (FlowJo LLC, Ashland, Oregon, USA).

Additionally, after the 4-hour treatment, the gel and supernatant were removed from some wells. The cells were then washed three times with cold PBS and fixed with 4% paraformaldehyde. Following fixation, the cell nuclei were stained with DAPI for 15 min and subsequently imaged using fluorescence microscopy.

#### Cellular cytotoxicity

The cytotoxic effects of our drug-loaded nanocomposite on 4T1 cells were evaluated using a Resazurin assay. The cell viability after treatment with MSN/5FU@CSH and MSN/EVE@CSH alone or in combination was compared to that of free-drugs alone or in combination, as well as controls, which we reported their results previously [43, 44]. Based on the LC of MSNs for each drug, we used 6 µg/ml MSN/EVE to achieve 0.5 µM EVE and 1 µg/ml MSN/5FU to achieve 3 µM 5FU, each added to 100 µl of CSH. Briefly, 2 × 10^4^ 4T1 cells were seeded on a 48-well plate. After an overnight incubation, 100 µl of pre-gel solution mixture was added to each well. The plate was gently shaken and incubated to allow the gel to form. After 48 h, the gel and the supernatant were removed and replaced with 180 µl of serum-free medium and 10 µl of Resazurin reagent solution. The plate was incubated for another 4 hours. Cell viability was calculated by measuring the absorbance at 520–570 nm using an ELISA reader and applying the following formula:

Viability = (OD test) / (OD control) × 100.

#### Apoptosis

The apoptosis of the cells was assessed using the FITC-Annexin V Apoptosis Detection Kit 1 according to the manufacturer’s instructions. The cells were seeded in the 6 well-plates at a density of 5 × 10^5^ cells per well and incubated overnight. Then, the cells were treated with different groups as described above in two wells per group. After 48 h, the cells were stained with Annexin V-FITC and PI (Propidium Iodide) and analyzed for apoptosis.

### In vivo assessment

All animal tests were conducted in accordance with the guidelines laid out by the Shahrekord University of Medical Sciences’ Laboratory Animal Center in Shahrekord, Iran (Ethical Code: IR.SKUMS.REC.1399.247). The antitumor efficacy of the different formulations was evaluated in BALB/c female mice bearing 4T1 tumors. The mice weighed 20 to 25 g and were divided into seven groups of eight mice. They were subcutaneously injected with 1 × 10^6^ 4T1 cells in the flank region. After about two weeks, when the tumors reached a volume of ~ 100 mm^3^, the mice in different groups received subcutaneous injections of 0.5 ml of PBS (control), MSN@CSH, MSN/5FU@CSH, MSN/EVE@CSH, and MSN/5FU-EVE@CSH in the right shoulder near the tumor region. The injections were repeated three times at seven-day intervals on days 1, 7 and 14. The tumor volume was measured daily for three weeks using a vernier caliper (Mitutoyo, Japan). On day 21, the mice were euthanized by an overdose of Ketamine-Xylazine and the tumor, kidney, liver, lung, and spleen tissues were harvested, weighed, and photographed for histological analysis. The tissues were fixed in 10% formalin for 72 h and then processed for paraffin embedding. Sections of 5 μm thickness were cut using a microtome and stained with Rapid-Hematoxylin and Eosin (Rapid-H&E). The sections were examined by a pathologist after imaging with light microscopy. The tumor volume and the tumor growth inhibition rate were calculated using the following equation:

$$V\left( {m{m^3}} \right) = 0.5{\text{ }}\left( {short{\text{ }}diamete{r^2} \times long{\text{ }}diameter} \right)$$

$$Inhibition{\text{ }}efficiency\% = \left[ {1 - \left( \begin{gathered}\left( {mean\,volume\,of\,treated\,groups} \right) \hfill \\/\left( {mean\,volume\,of\,control\,groups} \right) \hfill \\ \end{gathered} \right)} \right] \times 100$$

### Statistical analysis

All data interpret the results of three independent tests, shown as mean ± SD. Data were considered significant if *P* < 0.05. GraphPad Prism V8 was used to statistically analyze the data by one-way ANOVA and the Tukey post-hoc test.

## Results

### Characterizations of the nanocomposite

#### MSNs and CSH morphology

**Fig. 1:**
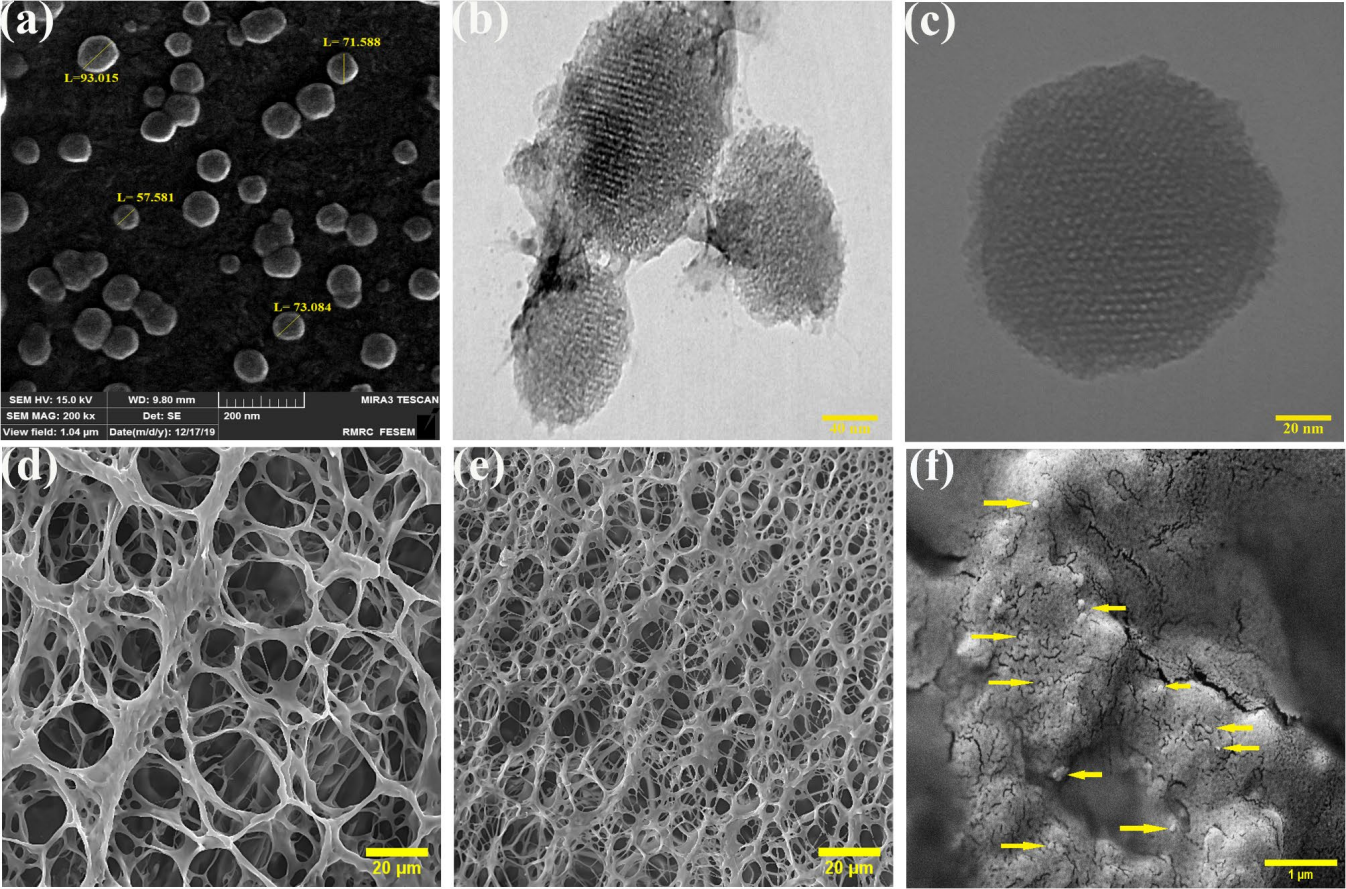
Morphological analysis of MSNs and CSH. **(a)** SEM image and **(b, c)** TEM images of MSNs; SEM images of **(d)** pure CSH, **(e)** β-GP crosslinked CSH and **(f)** β-GP crosslinked CSH with incorporated MSNs

The size and morphology of MSNs can vary depending on the synthesis method, conditions and different functionalizations [45–47]. In this study, MSNs were synthesized via the sol-gel method, resulting in spherical particles with a rough surface, an average size of 50–150 nm, exhibiting a honeycomb-like structure, as demonstrated by SEM and TEM images (Fig. 1a and b, and 1c). The obtained SEM images also revealed the porous scaffolds in both non-cross-linked (Fig. 1d) and cross-linked (Fig. 1e) CSH samples. Notably, the results demonstrated that the size of the holes in the CSH decreased from 10 to 20 μm to 5–10 μm in the cross-linked CSH, indicating that the cross-linking between CS molecular strands using β-GP molecules has led to the formation of a more complex network. Furthermore, the SEM images of cross-linked CSH containing MSNs (Fig. 1f) revealed the presence of dispersed nanoparticles within the denser hydrogel matrix. The morphological changes resulting from the cross-linking of CSH and the integration of MSNs are revealed by these observations, demonstrating the potential use of this nanocomposite for controlled drug delivery.

#### MSNs and CSH characterization

The drug loading properties of MSNs were evaluated after incorporating each therapeutic agent using the integration method. The results indicated high LE%, with 5-FU and EVE achieving 80.63 ± 3.11% and 87.01 ± 1.53%, respectively. The LC%, critical parameters for subsequent calculations, were 48.21 ± 1.16% and 8.73 ± 0.67% (w/w) for 5-FU and EVE, respectively.

**Fig. 2:**
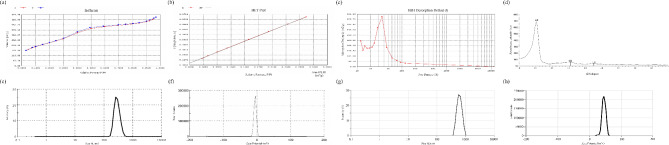
Nano-characterization of MSNs and CSH. **(a)** N_2_ adsorption-desorption isotherm plot of MSNs; **(b)** BET surface area analysis of MSNs derived from the isotherm data; **(c)** BJH pore size distribution plot of MSNs; (d) XRD pattern of MSNs, confirming the mesoporous structure; Particle size distribution and zeta potential of **(e, f)** MSNs and **(g, h)** β-GP crosslinked CSH with incorporated MSNs

The N_2_ adsorption-desorption is a commonly used technique to determine the surface area and pore size distribution of MSNs. In this study, the BET method was used to analyze the N_2_ adsorption-desorption isotherm of MSNs. The isotherm curve exhibits a Type IV shape classified as mesopores according to the IUPAC classification. (Fig. 2a). The surface area of 945.61 ± 6.48 m^2^/g was obtained (Fig. 2b) using the BET equation, which is a significant characteristic of mesoporous nanoparticles. Additionally, the BJH equation was used to estimate the pore size distribution of MSNs and a pore size of approximately 3.8 ± 0.2 nm was obtained (Fig. 2c) similar to previous studies that used mesitylene in the MSN synthesis process [48–51].

Furthermore, the XRD results in Fig. 2d provide valuable insight into the structural properties of the MSNs. The observed diffraction peaks at 2θ angles of 2.05°, 4.16°, and 5.43° correspond to periodic scattering from the mesostructure, which can be indexed to the [99, 109], and (200) reflections, respectively. The observed pattern is consistent with the P6mm space group, confirming the presence of an ordered hexagonal mesophase. This well-defined structural arrangement is crucial for applications in drug delivery and other biomedical fields, where porosity and stability play key roles [52, 53].

The DLS analysis of MSNs in this study revealed a maximum hydrodynamic diameter of 287 ± 17 nm (Fig. 2e) and a polydispersity index (PDI) of 0.182 ± 0.015, indicating acceptable dispersion. The PDI is a measure of the width of the size distribution, with a value closer to zero indicating a narrow distribution. The average PDI value suggests that the MSNs are relatively monodisperse, with a small variation in their sizes. Besides, the zeta potential measurement was also used to determine the surface charge of the MSNs by analyzing the electrostatic repulsion of particles in suspension. The zeta potential is influenced by the surface charge and ionic strength of the medium, and a higher absolute value indicates greater stability in suspension. The zeta potential of the MSNs in this study was − 7.18 ± 2.36 mV (Fig. 2f), which is attributed to the surface OH groups. Moreover, DLS and zeta potential analyses confirmed the successful incorporation of MSNs into CSH. The DLS results show an increase in the hydrodynamic size of the nanocomposite (694.05 ± 81.55 nm) compared to free MSNs (Fig. 2g), indicating the effective encapsulation of nanoparticles within the hydrogel matrix. Additionally, the zeta potential analysis reveals a shift from the negative surface charge of MSNs to the highly positive charge of the β-GP crosslinked CSH with incorporated MSNs (45.55 ± 3.63 mV, Fig. 2h). This suggests strong electrostatic interactions between the negatively charged MSNs and the positively charged chitosan hydrogel, facilitating stable incorporation. The highly positive zeta potential of the final nanocomposite further indicates improved colloidal stability, reducing nanoparticle aggregation and confirming the formation of a well-integrated MSN@CSH system.

#### FTIR spectra

**Fig. 3:**
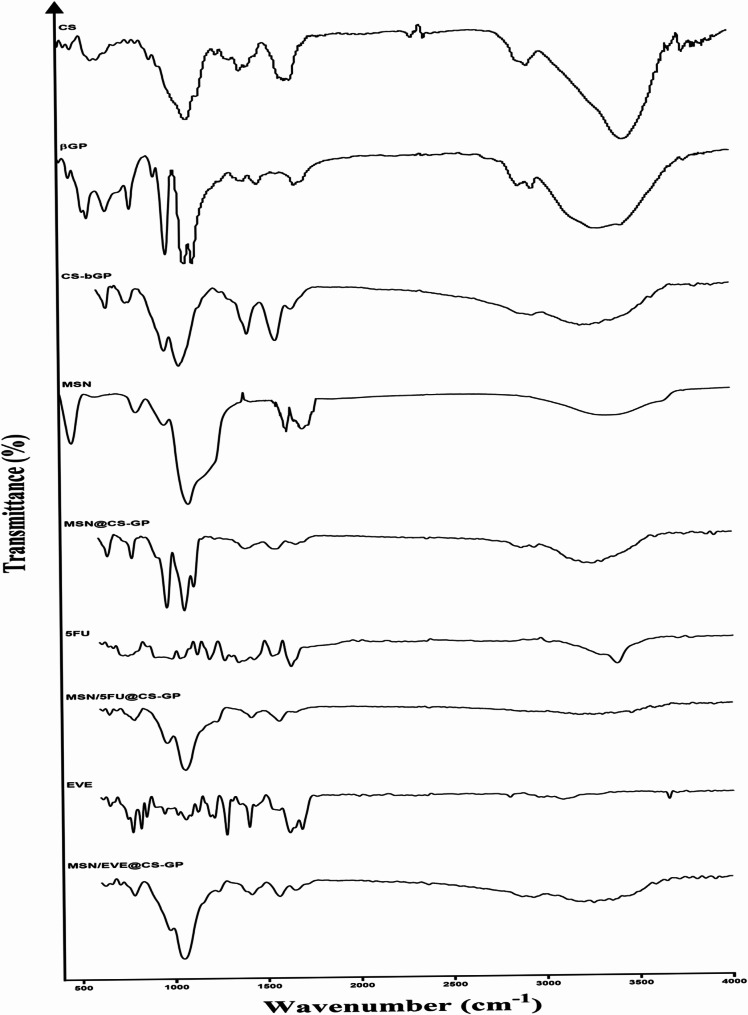
FTIR spectral analysis of different groups

In this study, the FTIR spectra provide insights into the successful loading of the drugs in the MSNs and the incorporation and interactions of drug-loaded MSNs within the hydrogel matrix (Fig. 3). The FTIR spectrum of pure CS shows characteristic peaks indicating its functional groups. The broad peak around 3200–3500 cm⁻¹ is attributed to the overlapping O-H and N-H stretching vibrations, indicative of hydrogen bonding. Amide I and II bands appear around 1650 cm⁻¹ (C = O stretching) and 1550 cm⁻¹ (N-H bending), respectively, which are related to the amide groups in CS. Additionally, peaks around 2800–2900 cm⁻¹ correspond to C-H stretching vibrations of the CS backbone [54, 55]. The β-GP spectrum shows characteristic peaks associated with phosphate groups and C-O-P stretching vibrations. Peaks around 1050–1150 cm⁻¹ are related to P = O stretching vibrations, while peaks around 850 cm⁻¹ correspond to C-O-P stretching vibrations. These peaks confirm the presence of β-GP in the hydrogel network [54, 56]. A shift in the C-O and C-O-C stretching groups in the CS and the β-GP was observed after crosslinking. The electrostatic interaction between the positive charge of the amino groups in CS and the negative charge of the phosphate groups in β-GP is the reason for these shifts. The interaction between them also results in the formation of a gel network, which is confirmed by the absence of further distinctive bands after gelation [56–58].

The FTIR spectrum of MSNs exhibits strong peaks around 1080 cm⁻¹, indicating the asymmetric stretching of Si-O-Si bonds. Additionally, a peak around 800 cm⁻¹ corresponds to Si-OH bending vibrations, and a peak around 460 cm⁻¹ corresponds to symmetric stretching vibrations of the Si-O-Si framework [48, 59]. When MSNs are incorporated into the CS-β-GP hydrogel (MSN@CS-GP), the FTIR spectrum shows significant changes. A broad peak at 3450 cm⁻¹ is observed due to the overlapping O-H and N-H stretching vibrations, indicating strong hydrogen bonding. Peaks at 1080 cm⁻¹ and 800 cm⁻¹ confirm the presence of Si-O-Si bonds from the MSNs. Additionally, peaks around 1040–1150 cm⁻¹ are attributed to the β-GP, confirming its presence in the hydrogel network. The amide I and II bands are slightly shifted, suggesting interactions between the CS matrix and the MSNs. The FTIR spectrum of free 5-FU shows characteristic peaks around 3400 cm⁻¹ due to N-H stretching vibrations. Peaks around 1676 cm⁻¹ and 1244 cm⁻¹ correspond to C = O and C-N stretching vibrations, respectively. When 5-FU is loaded into MSNs incorporated into the CSH (MSN/5FU@CS-GP), the FTIR spectrum shows significant changes. The characteristic peaks of 5-FU become less intense or shift slightly due to interactions with the silica matrix. New peaks appear corresponding to Si-O-Si stretching vibrations around 1080 cm⁻¹ and Si-OH vibrations around 800 cm⁻¹, which are characteristic of the silica framework. These changes in the FTIR spectrum provide evidence of successful drug loading, as the interaction between 5-FU and the silica nanoparticles alters the vibrational modes observed in the spectrum. The FTIR spectrum of free EVE shows characteristic peaks including C = O stretching vibrations around 1735 cm⁻¹, C-H stretching vibrations around 2920 cm⁻¹, C-O stretching around 1270 cm⁻¹, and N-H bending around 1550 cm⁻¹. When EVE is loaded into MSNs incorporated into the CSH (MSN/EVE@CS-GP), the FTIR spectrum shows less intense or slightly shifted peaks due to interactions with the silica matrix. New peaks at 1080 cm⁻¹ and 800 cm⁻¹ corresponding to Si-O-Si stretching and Si-OH vibrations, respectively, confirm the presence of the silica framework. These spectral changes indicate the successful loading of EVE into MSNs.

#### CSH gelation time and swelling ratio

Our results demonstrated that the incorporation of MSNs significantly enhances CSH’s mechanical and rheological properties. Specifically, the gelation time for free-CSH and MSN@CSH was observed to decrease from 527 ± 48 s to 112 ± 17 s, indicating an improved gelation behavior. The observed enhancement can be attributed to the surface silanol groups of MSNs which provide an interface for the adsorption of a significant number of CS molecules through their mesoporous structure. Additionally, the CSH and MSN/CSH both reached the highest weight and swelled status after 3.5 h, while the swelling ratio of the MSN@CSH was 3.3-fold higher than that of the CSH. The sturdy nature of MSNs enables them to withstand external pressure during swelling, loosening the gel network of the hydrogel. Their intricate web of pores functions like a network of tiny capillaries, effortlessly drawing in water. This unique property of MSNs transforms the chitosan hydrogel into a more adaptive and responsive system, enhancing its ability to absorb water and adjust to external conditions.

#### In vitro release profile of 5FU and EVE

**Fig. 4:**
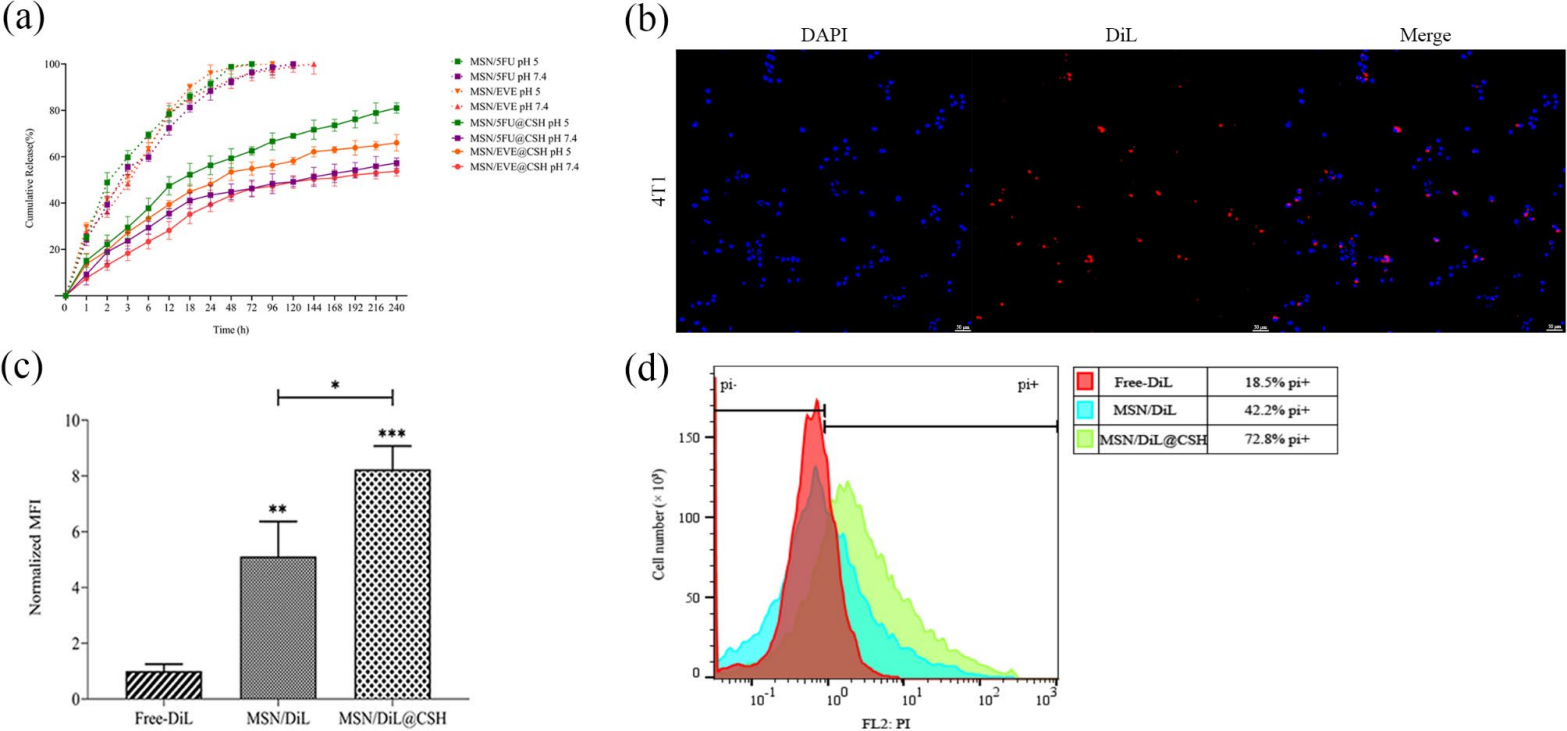
Drug release kinetics and cellular uptake. **(a)** Release profiles of drugs from the MSNs and MSN@CSH nanocomposite at different pH levels; **(b)** Fluorescent imaging showing cellular uptake of DiL-loaded nanoparticles in 4T1 cells stained with DAPI; **(c)** Mean fluorescence intensity of 4T1 cells, showing increased uptake of the nanosystems; **(d)** Flow cytometry results showing percentage of PI-positive cells, indicating cell viability and nanoparticle uptake. * P < 0.05, ** P < 0.01, *** P < 0.001

In this study, the release behavior of each drug from MSNs and MSN/drugs@CSH was investigated and indicated in Fig. 4a. On the one hand, the in vitro release profile of EVE and 5FU showed a pH-dependent release pattern, where the release of both drugs was higher at acidic pH compared to neutral pH in both MSN/drugs and MSN/drugs@CSH. This trend is expected because acidic pH can cause a collapse in CSH structure and increase the release of drugs [60–62], indicating the pH-responsive feature of our nanocomposite, which in acidic conditions could be beneficial in cancer treatment, as tumors have an acidic microenvironment that can enhance drug release and efficacy [63]. On the other hand, our results showed that the release profile of MSN-encapsulated drugs with a pore size of 3 nm was slow but continued just for 4 days at pH 5, while just about 70% of the loaded 5FU and 60% of loaded EVE were released from the MSN/drug@CSH in 10 days at pH 5.

### Cellular assessment

#### Cellular uptake

For investigating the cellular uptake, we used a fluorescent dye (DiL)to be loaded in the nanosystem and can be detectable. MSN/DiL@CSH and free DiL have been incubated for 4 h with 4T1 cells. Images from the fluorescent microscope showed that the formulated DiL was internalized into the cells and remained chiefly in the cytoplasm (Fig. 4b). The quantity of internalized particles in 4T1 cells was assessed by using the mean fluorescent intensity (MFI) of 100,000 cells (Fig. 4c) and the flow cytometry histogram plots showing the percentage of the PI-positive cells as determined by flow cytometry (Fig. 4d). Our results show that compared to DiL, the fluorescence intensity of internalized MSN/DiL and MSN/DiL@CSH increased 5- and 8-fold, respectively (*p* < 0.0001, Fig. 4e). The fact that MSN/DiL was internalized into cells and remained in the cytoplasm is promising for the use of MSN as a drug delivery platform, as drugs loaded into MSNs could be more efficiently taken up by cells compared with free drugs and remain localized in the cytoplasm [64, 65]. Besides, the significant increase in uptake of MSN/DiL@CSH is due to the CS’s positively charged nature, which imparts a charge on the surface of nanoparticles, enabling their interaction with the negatively charged cell membrane and facilitating their entry into cells. Upon exposure to the acidic environment of cancer cells, it is possible that the MSNs become coated with a layer of CS as they are released from the CSH. The degree of CS coating on the nanoparticles can vary, ranging from complete coverage to partial or no coverage at all [66, 67]. This multifaceted approach enables not only the regulated release of nanoparticles and drugs but also protects them from degradation and aggregation.

#### Cellular cytotoxicity and drug synergism

**Fig. 5:**
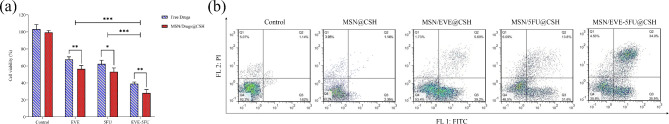
In vitro efficacy of the nanosystem in 4T1 breast cancer cells. **(a)** Cell viability of 4T1 cells treated with various formulations, highlighting the cytotoxic effect of the nanocomposite-based co-delivery system; **(b)** Flow cytometry analysis showing the rate of apoptosis in treated 4T1 cells. *** P < 0.001

The results of the cytotoxicity in our study demonstrated that CSH encapsulated drug-loaded MSNs had significantly higher cytotoxic effects than free EVE, 5FU, and EVE-5FU combination at equivalent concentrations (Fig. 5a). This finding suggests that the use of this nanocomposite platform as a drug carrier can improve the therapeutic efficacy of drugs by enhancing their uptake and accumulation in cells. The higher cytotoxicity of MSN/EVE@CSH and MSN/5FU@CSH compared to free drugs could be attributed to the sustained release of drugs from MSN-CS nanocomposite, which could lead to a higher concentration of drugs in cells over time. The lack of significant cytotoxicity observed after 48 h of treatment with free-MSNs and free-MSN@CSH suggests that the nanocomposite does not play a significant role in cytotoxicity. The synergistic effect of MSN/EVE-5FU@CSH therapy was proved by the Chou-Talay method using Compusyn Software (S1) and the combined treatment resulted in lower cell viability compared to single drug-loaded MSNs (*P* < 0.0001). This finding suggests that the co-delivery of EVE and 5FU loaded separately in MSNs and their incorporation in the hydrogel not only increased the synergism effects of their combination but also enhanced their therapeutic efficacy by the increased cellular uptake and controlled release. It is due to the enhanced targeting of different pathways involved in cancer progression as we previously demonstrated for the free EVE-5FU combination [43].

#### Apoptosis induction

The results of the flow cytometry and FACS analysis demonstrated that treatment with MSN/EVE-5FU@CSH induced a significantly higher level of apoptosis in 4T1 cells compared to treatment with each formulated drug individually (Fig. 5b). Specifically, the MSN/EVE@CSH and MSN/5FU@CSH induced apoptosis at approximately 42%, while treatment with MSN/EVE-5FU@CSH induced apoptosis at about 62.13 ± 2.40%, respectively. Furthermore, MSN/EVE-5FU@CSH induced significantly more apoptosis compared to free EVE-5FU, which we evaluated previously at about 39.75 ± 2.11% [43]. The observation that combinatorial treatment using our MSN@CSH nanoformulation as a delivery system could induce more late apoptosis in 4T1 cells than early apoptosis is interesting and suggests that this nanocomposite also can improve the therapeutic efficacy of drugs by targeting different pathways involved in cancer cell death [68–71] by improving the uptake and synergistic effects of drugs [68, 72–75].

### In vivo assessment

To examine the ability of our system to deliver anticancer drugs and suppress tumor growth in mice, we used Balb/C mice as a breast cancer model. The in vivo degradation of the CSH was analyzed by our team in the previous study [44] and the results showed that the CSH degraded after 9 days, hence, we prepared a suitable amount of nanoparticles containing the required concentration of each drug for a week. The concentration of drugs was determined based on previous experiments using 5FU [76, 77] and EVE [78–80]. Briefly, we administered 0.5 ml of the nanocomposite formulation subcutaneously three times at seven-day intervals. The nanocomposite for each group of treatment contained about 3.5 mg/ml of MSN/EVE and 0.7 mg/ml of MSN/5FU to achieve an equivalent EVE and 5FU concentration of 15 mg/kg/week, respectively. PBS and free MSN@CSH were administrated as control groups.

#### Body weight and tumor size

**Fig. 6:**
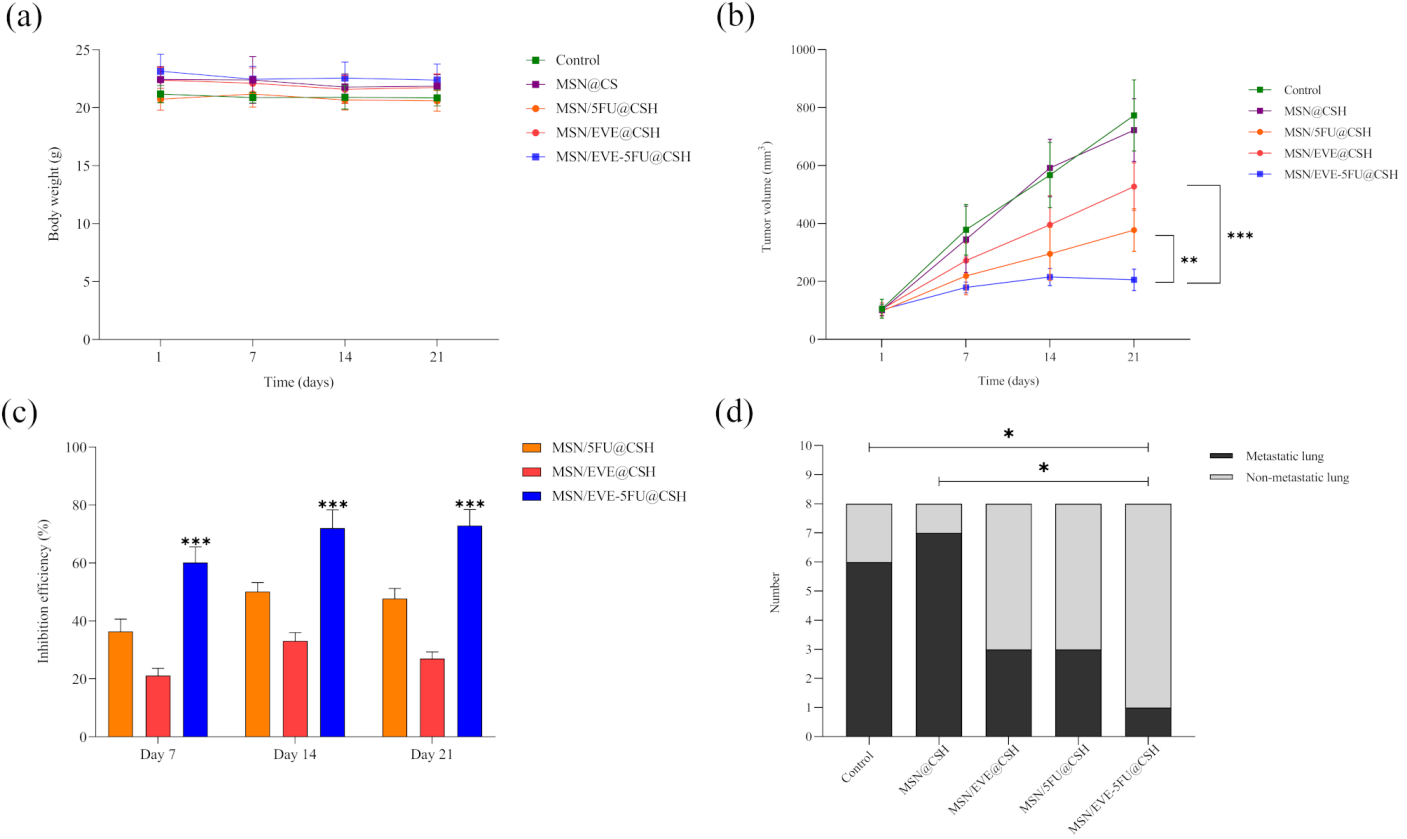
In vivo evaluation of the nanosystem in a BALB/c mouse breast cancer model. **(a)** Body weight changes and **(b)** Tumor volume changes in mice during and after the treatment period. **(c)** Tumor growth inhibition efficiency for each treatment group. **(d)** Number of mice with lung metastases in each group after treatment. * P < 0.05, ** P < 0.01, *** P < 0.001 (*n* = 8)

Throughout the study, no significant body weight loss was observed in any of the treated mice compared to the control groups (Fig. 6a). During the injection period, none of the mice displayed severe toxicity symptoms such as shivering, inactivity, severe tail necrosis, ataxic gait, or a sudden decrease in body weight. As shown in Fig. 6b, Tukey’s post hoc test revealed a significant difference in average tumor size on each of the investigated days (*p* < 0.01). Notably, the average tumor size on the 21st day was significantly smaller than on the first, seventh, and fourteenth days. In the control and free MSN@CSH groups, tumors continued to grow rapidly, reaching average volumes of approximately 750 mm³ by the 21st day, indicating that the free MSN@CSH did not affect tumor growth in mice.

In contrast, treatment with MSN/5FU@CSH and MSN/EVE@CSH resulted in significantly slower tumor growth (*P* < 0.0001), with final average tumor volumes of 377 ± 73.54 mm³ and 527.08 ± 82.00 mm³, respectively, by day 21. Remarkably, in the combination treatment group (MSN/5FU-EVE@CSH), tumor growth ceased after the second injection, and tumors remained relatively small throughout the treatment period, with very small final average tumor volumes of 195.41 ± 37.05 mm³ (*P* < 0.0001). Furthermore, the combination therapy led to a significantly greater reduction in tumor size compared to both MSN/EVE@CSH (*P* < 0.0001) and MSN/5FU@CSH (*P* < 0.01). The tumor growth inhibition results (Fig. 6c) further underscored the efficacy of the combinatorial treatment, showing a significant difference (*P* < 0.0001) with 72.93 ± 5.49% inhibition, compared to 27.01 ± 2.30% with MSN/EVE@CSH and 47.74 ± 3.48% with MSN/5FU@CSH.

#### Metastasis

**Fig. 7:**
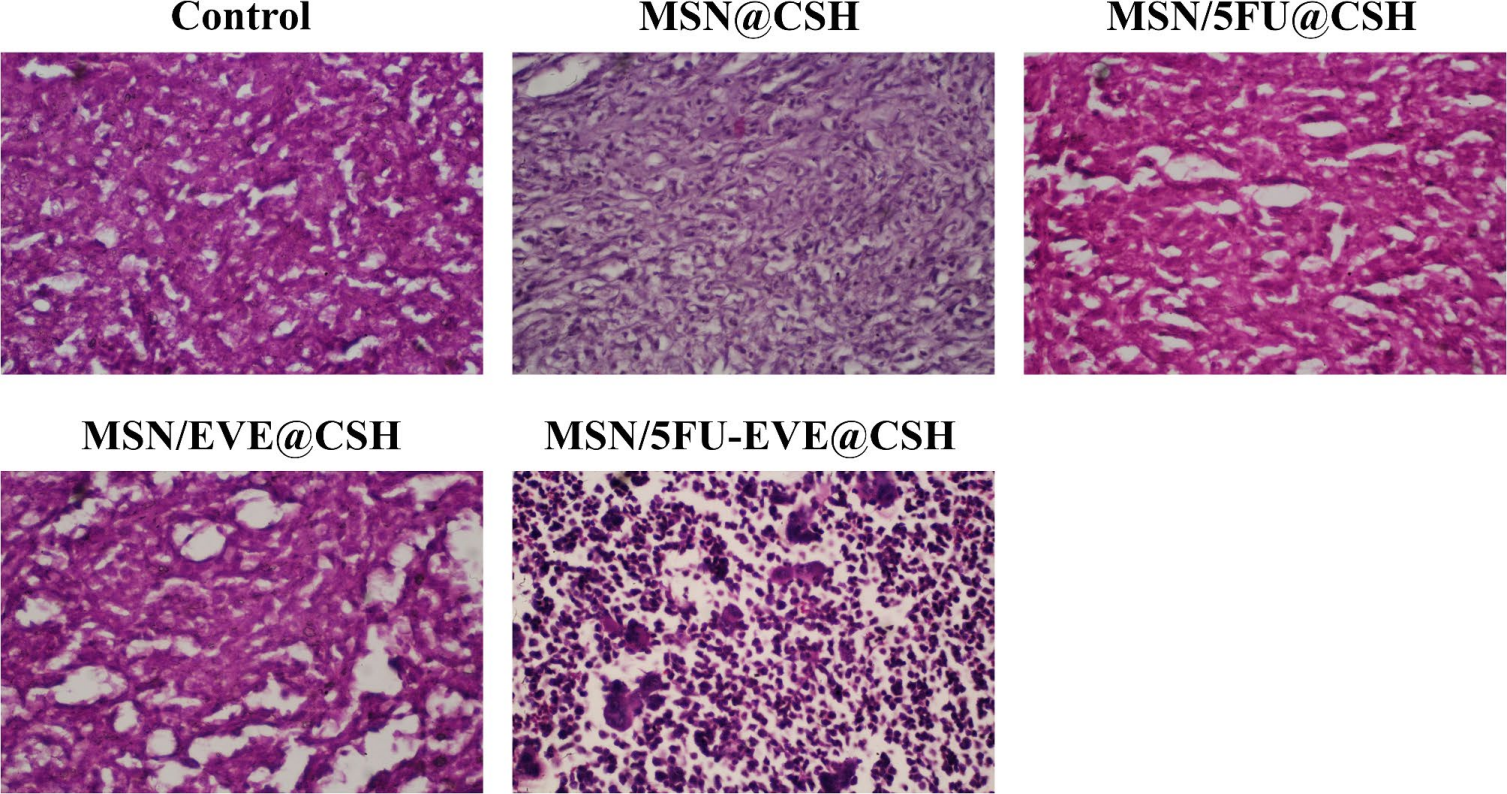
Histological analysis of tumor tissues from different treatment groups stained with hematoxylin and eosin (H&E). The control groups show dense tumor tissue with minimal necrosis. The final treatment group demonstrates extensive tumor cell death and necrosis, highlighting the enhanced therapeutic efficacy of the combination therapy

Metastasis was also investigated by visual observance of the tissues and light microscopy through H&E staining across various tissues, revealing a significant correlation with lung metastasis among the studied groups. In the control and MSN@CSH groups, lung metastasis was present in 13 out of 16 cases. In contrast, only 3 cases of lung metastasis were observed in both the MSN/5FU@CSH and MSN/EVE@CSH groups. Notably, treatment with MSN/5FU-EVE@CSH resulted in a significant reduction in lung metastasis, with only one case detected, showing a statistically significant difference compared to the control groups (*P* < 0.05, Fig. 6d). The histological analysis of tumor tissues presented in Fig. 7 demonstrates the significant therapeutic effect of the MSN/5FU-EVE@CSH nanosystem compared to other treatment groups. The control group exhibits dense tumor tissue with minimal necrosis, indicating unchecked tumor proliferation. Treatment with MSN@CSH alone shows slight structural alterations but lacks substantial tumor suppression. Both MSN/5FU@CSH and MSN/EVE@CSH groups exhibit moderate tumor disruption, suggesting partial effectiveness of monotherapy with either 5FU or EVE. In contrast, the MSN/5FU-EVE@CSH group reveals extensive tumor cell death, characterized by significant nuclear fragmentation and increased areas of necrosis. This pronounced therapeutic response highlights the increased anticancer effect of the dual-drug-loaded nanosystem, likely due to enhanced drug co-delivery, improved bioavailability, and sustained release at the tumor site.

## Discussion

Our previous investigations into the synergistic effects of 5FU and EVE have demonstrated significant anti-cancer efficacy [43]. However, the co-delivery of these agents via nanoplatforms has yet to be explored. In this study, we present a novel synergistic co-delivery strategy utilizing stimuli-responsive CSH-based nanoplatforms for the simultaneous administration of MSNs loaded with 5FU and EVE in the treatment of breast cancer.

We selected MSNs for drug delivery due to their favorable properties. Key parameters such as surface area and pore size distribution of MSNs significantly influence their performance across various applications. The high surface area of MSNs facilitates efficient drug loading, functionalization, and delivery, prompting researchers to enhance this parameter for improved efficacy. Recent studies have achieved the synthesis of hollow MSNs with a remarkable surface area of 1496 m²/g using a triple surfactant-assisted soft-templating method [81], whereas most studies report surface areas ranging from 700 to 1000 m²/g [82], particularly those employing the sol-gel method [83, 84].

To contextualize our findings within the existing literature, while numerous studies have established that MSNs loaded with 5FU exhibit a slow-release profile and enhanced cytotoxicity against cancer cells compared to free 5FU [85–89], similar findings concerning EVE have not been documented in prior research. Nevertheless, we can draw parallels between EVE-loaded MSNs and those encapsulating RAPA [90–94] due to the structural and molecular weight similarities. Such studies have corroborated our findings regarding loading properties, controlled drug release, and increased cytotoxicity compared to their free-drug counterparts. For example, recent research highlighted that RAPA is optimally retained in mesopores that slightly exceed its molecular dimensions. MSNs with smaller pore sizes exhibit slower drug release profiles, particularly for this hydrophobic small molecule, attributed to increased solvent accessibility in larger-pored MSNs [91]. This observation aligns with our results, as our MSNs featured a small pore size of 3.6 nm, which affects the release kinetics of the loaded drugs and is crucial for determining the selectivity of MSNs toward different molecules. Furthermore, the higher loading capacity percentage (LC%) of our MSNs for both 5FU and EVE, in comparison to previous studies employing similar or modified MSNs [93, 95, 96], may be ascribed to the large surface area and small pore size of our MSNs. In contrast, a recent study reported an equal LC% for SPION-MSN-NH2 with a pore size of 3 nm for 5FU [97] that may stem from the prolonged stirring time of 48 h, and MSN: drug ratio which has been demonstrated to positively influence LC% [98].

Despite the considerable volume of preclinical studies conducted, only a limited number of silica-based nanomaterials have received authorization from the FDA for clinical trials. The transition from in vitro studies to in vivo applications presents a formidable challenge, as several critical factors must be taken into account. These include opsonization, the enhanced permeability and retention (EPR) effect, nanoparticle transport within the bloodstream, the formation of a protein corona, and the rapid clearance of nanoparticles by immune cells. Accurately replicating these complexities in in vitro models proves to be a significant hurdle [99]. Therefore, it is imperative to develop and implement strategies aimed at overcoming these obstacles to facilitate the advancement of silica-based nanomaterials in clinical settings.

Studies have shown that CSHs are suitable tools for the delivery of different biomacromolecules [75, 100], however, natural polysaccharide-based hydrogels, while biocompatible, often face challenges such as drug leakage and rapid burst release due to their high hydrophilicity and weak mechanical strength. By adding MSNs, CSHs have shown that can address these limitations by improving the structural integrity of the gel, which offers better protection for encapsulated bioactive agents. The MSNs not only reinforce the mechanical properties of the hydrogel but also enable a more controlled and sustained release of therapeutic compounds, reducing the risk of premature drug release and enhancing overall treatment efficacy [101, 102]. The dual stimulatory-responsive system of the MSN@CSH nanocomposite has exhibited impressive outcomes in regulating drug release rates, enhancing cellular uptake, and improving therapeutic effects in vitro and in vivo. The solubility and hydrophobicity of CSH are influenced by protonation, which can result in hydrogel fragmentation. The degradation and release rates can also be modulated by varying environmental pH and hydrogel characteristics. Controlled release of the encapsulated material can thus be achieved by exploiting the degradation of CSH in acidic conditions, liberating contents into the surrounding environment as the hydrogel degrades [103]. Although a slower release could raise concerns about insufficient drug concentration, after the full degradation of the hydrogel (approximately 9 days), the maximum amount of drug-containing nanoparticles becomes accessible, maintaining an effective therapeutic concentration. This slow drug release offers a controlled and sustained delivery, ensuring prolonged drug availability at the tumor site while minimizing systemic fluctuations. This also helps to continuously expose tumor cells to therapeutic levels of the drugs, potentially reducing resistance and enhancing treatment efficacy. These modifications in our nanocomposite were evident in the results of scanning electron microscopy (SEM), zeta potential, Fourier-transform infrared spectroscopy (FTIR), gelation time, and swelling ratio assays.

The SEM images revealed that following the incorporation of MSNs, the hydrogel structure exhibited increased density with significantly smaller pores after crosslinking with β-GP, creating a denser environment around the MSNs. This is attributed to the distribution of MSNs on the hydrogel surface, serving as crosslinking points to enhance the hydrogel network’s density [102]. Zeta potential is another crucial factor in characterizing nanosystems, indicating the surface charge of the platform. We observed a major transition from the negative zeta potential of MSNs to the highly positive surface charge of the MSN@CSH composite. Positively charged nanosystems may encounter challenges in drug delivery through the bloodstream due to potential interactions with negatively charged cell membranes and plasma proteins. Such interactions can lead to the formation of nanoparticle-protein aggregates, diminishing the system’s targeting efficiency and its ability to home in tumor microenvironments [104]. However, despite these limitations, a positive surface charge is more conducive to localized delivery approaches, such as subcutaneous or intratumoral injections, where proximity to the tumor site mitigates off-target interactions and enhances therapeutic efficacy. First, a positively charged surface improves interactions between nanoparticles and negatively charged cell membranes, facilitating cellular uptake through electrostatic attraction. This interaction is particularly critical for targeted delivery to specific tissues or cells, ensuring that therapeutic agents reach their intended destination with higher efficiency [105]. Furthermore, nanoparticles exhibiting a positive zeta potential demonstrate enhanced stability in biological fluids, reducing the risk of aggregation that could compromise their circulation time and bioavailability [106]. Additionally, the positive charge can aid in the protection and controlled release of encapsulated drugs, particularly in acidic environments like tumor tissues, where it may contribute to EPR effects, leading to more effective drug accumulation at the target site [107]. Moreover, a notable increase in uptake of the nanocomposite compared to free MSNs is attributed to the positively charged nature of CS, which imparts a charge on the surface of nanoparticles, facilitating their interaction with negatively charged cell membranes and promoting cellular entry. Upon exposure to the acidic environment of cancer cells, it is conceivable that the MSNs become enveloped in a dense layer of CS as they are released from the CSH. The extent of CS coating on the nanoparticles can vary from complete coverage to partial or no coverage. This phenomenon has been documented in prior studies [66, 67], enhancing the interaction between our designed system and the cell membranes of cancer cells, resulting in heightened uptake and efficiency.

Concerning gelation time, studies have indicated that the gelation mechanism of β-GP-CS is influenced by various factors, including the concentration of each component, final pH, and temperature [57, 108–111]. Nonetheless, temperature emerges as the most critical factor affecting gelation time, with increases in temperature corresponding to decreased gelation time [108, 109]. The influence of temperature on the gelation time of CS has been documented in various publications, which report accelerated gelation at elevated temperatures and β-GP concentrations [75, 112, 113]. The acceleration of the gelation process is attributed to enhanced CS–CS interactions and reduced mobility of polymer chains, alongside increased hydrogen bonding interactions at higher temperatures. Consequently, this elevates the entropy of the CS solution, facilitating proton release from CS. The CS chains are subsequently brought closer together, resulting in the gelation of the CS gel [112]. Consistent with our results, studies have indicated that incorporating MSNs into the hydrogel decreases gelation time, leading to a faster gelation rate; notably, the time required for complete gelation of the MSN@CSH is significantly shorter than that for CSHs alone. This phenomenon is attributed to the abundant silanol groups on the MSNs’ surface, which interact with and absorb a substantial number of CS molecules [103], creating a dense meshwork of CS chains around the MSNs and potentially providing steric stabilization to prevent phase separation and particle aggregation [114]. Additionally, the rigid structure of MSNs may also contribute to strengthening the CS network [103]. These changes are reflected in the shifts observed in C-O and C-O-C stretching groups in the CS and β-GP following crosslinking, as confirmed by FTIR results. The electrostatic interaction between the positive charge of the amine groups in CS and the negative charge of the phosphate groups in β-GP accounts for these shifts. The interaction between these components results in the formation of a gel network, as evidenced by the absence of distinctive bands after gelation [56–58]. Furthermore, our results indicated that the incorporation of MSNs into the β-GP-crosslinked CSH produced significant alterations in the FTIR spectrum, suggesting interactions between the CS matrix and the MSNs.

In 2010, silica nanoparticles were first incorporated into CSHs, where researchers investigated their use as sustained-release vaccine carriers in CSHs. Their study found that CS gels containing ovalbumin (OVA)-loaded nanoparticles and the adjuvant Quil A (QA) exhibited a significantly greater ability to induce CD4 + T cell proliferation in vivo compared to CS gels containing soluble OVA and QA [115].

Subsequently, in another study by Zhu et al., the release of gentamicin (GC) and bovine serum albumin (BSA) from MSNs embedded in CSHs was investigated. The results indicated that the release rate was modulated by the crosslinking density of the hydrogels and the presence of MSNs [103]. Their co-delivery approach, where BSA was loaded into MSNs and mixed with CS and GC before the addition of β-GP, resulted in significantly enhanced chondrocyte proliferation and maintenance of chondrocyte phenotype, showing potential for drug delivery. Notably, the CSH cross-linked with β-GP gelled in 5 min at 37 °C, while gelled immediately after the addition of MSN. In our study, we observed a slower gelation time of 2 min, which could be attributed to differences in β-GP and MSN concentrations. Despite these variations, the gelation time remains acceptable for in vivo applications. Our swelling ratio results also showed similar changes. Additionally, their study showed that only 22% of BSA was released from the nanocomposite after 7 days, consistent with our findings, where drug release from the MSN/drug@CSH was significantly slower than from free or MSN-loaded drugs.

Later, in 2016, Hu et al. designed hybrid composite hydrogel beads using three different polysaccharides (alginate, hyaluronic acid, and CS) [101]. They demonstrated that the incorporation of MSNs into the hydrogel structure, crosslinked with CaCl2, enhanced the physical crosslinking networks within the hydrogel. This was attributed to the absorption effects of the MSNs, resulting in a decreased swelling ratio due to the more immobilized and restricted proportion of MSNs within the hydrogel’s polymer network. Their platform exhibited favorable loading properties, with BSA release kinetics indicating that the composite could protect BSA from external denaturation, leading to slow and sustained release in a predictable manner. However, they noted that concentrations of MSNs exceeding 6 mg/ml resulted in toxicity, which is a critical consideration. In our system, the incorporated MSN concentration was less than 4 mg/ml, exhibiting no toxic effects, as evidenced by our cell viability assays and histological examination of essential tissues.

In 2019, Xia et al. found that the combination of gold-coated porous silicon nanoparticles with CS held promise for localized chemo-photothermal therapy in cancer treatment [116]. The injectable nanocomposite hydrogel was composed of porous silicon nanoparticles embedded in β-GP-crosslinked CS. The shifts in the FTIR results mirrored those observed in our study, with peak intensities of phosphate groups stretching at 961 cm-1 decreasing, alongside reductions in peak intensities at 1354, 1441, 2840, and 2909 cm-1, resulting in a decreased ratio of amide I and II. Similar to our findings, they attributed the gelation process of the CS/β-GP precursors to synergistic molecular interactions, including hydrogen bonding, electrostatic, and hydrophobic interactions, which is consistent with our findings. Their gelation time was approximately 6 min at 37.0 °C. Furthermore, the CSH served as an outer layer, protecting the photothermal activity of the inner layer. However, in contrast to our findings, the in-situ degradation of their developed composite occurred over approximately 14 days, resulting in slight degeneration of liver hepatocytes, with no significant morphological changes observed in other tissues.

A subsequent study in 2022 designed an innovative dual-hydrogel platform responsive to both pH and glutathione for the co-delivery of cytarabine (loaded in NH2-MSNs incorporated within a sodium hyaluronate hydrogel) and methotrexate (loaded in CS and oxidized sodium carboxymethyl cellulose hydrogel) [117]. Their results indicated that incorporating MSNs enhanced the release controllability of the hydrogels, rendering them dual stimulatory-responsive. Consistent with our findings, their in vitro cell viability assays demonstrated that the nanosystem was non-toxic, biocompatible, and biodegradable, with enhanced anticancer effects observed when drugs were co-delivered for combination therapy compared to free or single treatments in cancer cells. This team further developed another hydrogel-based nanocomposite featuring disulfide-presented MSNs embedded in carboxymethyl CS and oxidized pullulan, resembling the dual stimulatory-responsive nanocomposite aimed at targeting folic acid and delivering methotrexate to hepatoma cancer cells [118]. This composite exhibited excellent biocompatibility and significantly enhanced growth inhibition against human hepatoma SMMC-7721 cells.

## Conclusion

In conclusion, this study demonstrates that the integration of MSNs into a stimuli-responsive CSH presents a promising strategy for the co-delivery of anti-cancer agents, enhancing therapeutic efficacy while minimizing side effects. This multifaceted approach enables not only the regulated release of nanoparticles and drugs but also protects them from degradation and aggregation, which enhances the therapeutic efficiency of our co-delivery strategy, improves the synergism and combinatorial treatment effect by preventing the drug interactions and reduced loading properties of the nanoparticles where the drugs are co-loaded as well as reducing the drug dosage and injections for better health outcomes and patient comfort. These findings suggest that this nanocomposite delivery platform offers a versatile and effective approach for future cancer treatments, potentially addressing the limitations of current co-delivery and combination therapies and paving the way for clinical translation.

## Electronic supplementary material

Below is the link to the electronic supplementary material.

Supplementary Material 1

## Data Availability

The datasets used and analyzed during the current study are available from the corresponding author upon a reasonable request.

## Abbreviations

5FU: 5–Fluorouracil
BSA: Bovine Serum Albumin
CS: Chitosan
CSH: Chitosan Hydrogel
DiL: 1,1’–Dioctadecyl–3,3,3’,3’–Tetramethylindocarbocyanine Perchlorate
DAPI: 4′,6–Diamidino–2–Phenylindole
DLS: Dynamic Light Scattering
DOX: Doxorubicin
EPR: Enhanced Permeability and Retention
EVE: Everolimus
FTIR: Fourier Transform Infrared Spectroscopy
GC: Gentamicin
H&E: Hematoxylin and Eosin
HUVECs: Human Umbilical Vein Endothelial Cells
LC%: Loading Capacity Percentage
LE%: Loading Efficiency Percentage
MFI: Mean Fluorescence Intensity
MSNs: Mesoporous Silica Nanoparticles
OVA: Ovalbumin
PBS: Phosphate Buffered Saline
PDI: Polydispersity Index
PI: Propidium Iodide
PTX: Paclitaxel
QA: Quil A
RAPA: Rapamycin
RPMI: 1640–Roswell Park Memorial Institute Medium
SEM: Scanning Electron Microscopy
TEM: Transmission Electron Microscopy
β: GP–β–Glycerophosphate
