# Supplementary material for: A novel approach for the co-delivery of 5-fluorouracil and everolimus for breast cancer combination therapy: stimuli-responsive chitosan hydrogel embedded with mesoporous silica nanoparticles
Source: J Transl Med. 2025 Mar 31;23:382. doi: 10.1186/s12967-025-06396-4 (PMC11956229; doi:10.1186/s12967-025-06396-4)
Supplement: Supplementary file 1 — Supplementary Material 1 [file 12967_2025_6396_MOESM1_ESM.docx]

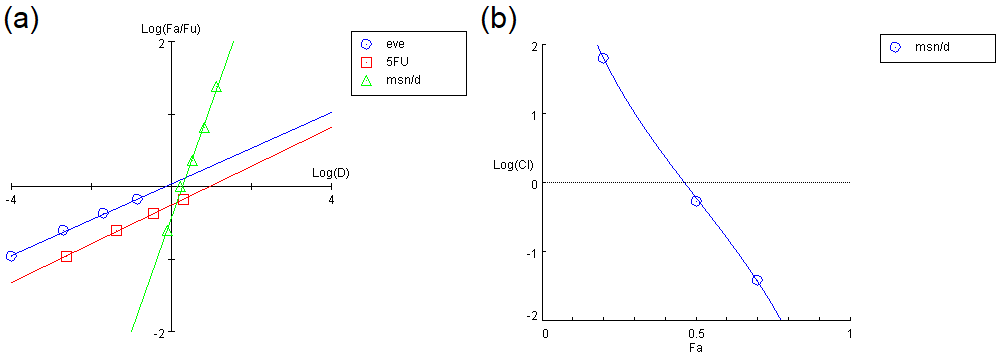
Figure 1: Median-Effect Plot (a) and Logarithmic Combination Index Plot (b) produced by the Compusyn Software based on the constant ratio Chou-Talay method.

| Drug Combination | Combination Ration | CI values at inhibition of | | | |
| --- | --- | --- | --- | --- | --- |
|  |  | 50% | 75% | 90% | 95% |
| 5FU + EVE | 1:6 | 0.599 | 0.015 | 4.27 × 10^-4^ | 3.74 × 10 ^-5^ |

Table 1: Combination index at different growth inhibition of 4T1 cells.
